# LATE DIAGNOSIS OF CONGENITAL SYPHILIS: A RECURRING REALITY IN WOMEN AND CHILDREN HEALTH CARE IN BRAZIL

**DOI:** 10.1590/1984-0462/;2018;36;3;00011

**Published:** 2018

**Authors:** Ana Laura Mendes Becker Andrade, Pedro Vitor Veiga Silva Magalhães, Marília Magalhães Moraes, Antônia Teresinha Tresoldi, Ricardo Mendes Pereira

**Affiliations:** a Universidade Estadual de Campinas, São Paulo, Brasil.

**Keywords:** Child, Congenital syphilis, Primary health care, Sentinel surveillance, Criança, Sífilis congênita, Atenção primária à saúde, Vigilância de evento sentinela

## Abstract

**Objective:**

To describe a case of congenital syphilis with a late diagnosis and identify missed opportunities at diverse phases/levels of healthcare, which led to late diagnosis.

**Case description:**

Boy, 34 days of life, referred from a basic healthcare unit to a tertiary hospital due to enlarged abdominal volume and progressive jaundice for 2 weeks, fecal hypocholia, hepatosplenomegaly, anemia, low platelet count and elevated liver enzymes. At physical examination, the infant presented with erythematous-exfoliative lesions on the palms and soles, macular rash in the inguinal region, ascitis, palpable liver 5 cm below the right costal margin and a palpable spleen 3 cm from the left costal margin. Infant serology: reactive CMIA (chemiluminescent microparticle immunoassay), VDRL (Venereal Diseases Research Laboratory) 1:1024 and reactive TPHA (*Treponema pallidum* Hemagglutination). Maternal serology: reactive CMIA and TPHA, VDRL 1:256. Radiography of the long bones showed symmetric periostitis, periosteal thickening, and lucent bands in the femur, humerus, ulna and tibia. After treatment with crystalline penicillin, the infant showed clinical and laboratory improvement, receiving hospital discharge at the 18^th^ hospitalization day.

**Comments:**

This case shows that congenital syphilis is occasionally diagnosed late as a result of failed strategies to prevent this disease, both in the basic and secondary/tertiary levels of care. The application of interventions recommended by the Ministry of Health and identification of the situation in which there is ineffective implementation of these measures are important to assess routine care in all levels of healthcare and diverse units responsible for newborn and infant health care.

## RESUMO

**Objetivo:**

Descrever um caso de sífilis congênita com diagnóstico tardio e identificar as oportunidades perdidas nas diversas fases/níveis da atenção à saúde, que retardaram a realização do diagnóstico.

**Descrição do caso:**

Menino, 34 dias de vida, encaminhado da Unidade Básica de Saúde a um hospital terciário por apresentar aumento do volume abdominal e icterícia progressiva há 2 semanas, hipocolia fecal, hepatoesplenomegalia, anemia, plaquetopenia e elevação de enzimas hepáticas. Ao exame físico, apresentava lesões eritemato-descamativas nas mãos e nos pés e exantema macular em região inguinal, presença de ascite, fígado palpável a 5 cm do rebordo costal direito e baço palpável a 3 cm do rebordo costal esquerdo. Sorologia do lactente: CMIA (quimioluminescência de micropartículas) reagente, VDRL (*Venereal Diseases Research Laboratory*) 1:1024 e TPHA (*Treponema pallidum Hemaglutination*) reagente. Sorologia materna: CMIA e TPHA reagentes, VDRL 1:256. Radiografia de ossos longos mostrava periostite simétrica; levantamento periosteal; e bandas metafisárias lucentes em fêmures, úmeros, ulnas e tíbias. Após tratamento com penicilina cristalina, apresentou melhora clínica e laboratorial, recebendo alta no 18º dia de internação.

**Comentários:**

Este caso mostra que ainda ocorre diagnóstico tardio de sífilis congênita por falhas nas estratégias de prevenção dessa doença, tanto na atenção básica quanto nos níveis secundário e terciário. A aplicação das intervenções preconizadas pelo Ministério da Saúde e a identificação das situações em que ocorrem falhas na sua execução são importantes para a avaliação da assistência de rotina em todos os níveis de atenção e nas diversas unidades responsáveis pelo cuidado do recém-nascido e do lactente jovem.

## INTRODUCTION

Syphilis is a systemic, preventable infectious disease; when not treated early, it may evolve to a chronic stage with irreversible sequelae. It is sexually and vertically transmitted, and rarely via blood transfusion. The notification for this disease is mandatory: Ordinances 542/MS^1^ and 33/MS/SVS.^2^

Congenital syphilis (CS) corresponds to the infection of the fetus with *Treponema pallidum*, being transmitted through placental transfer at any moment of the pregnancy, regardless of the clinical stage of the disease in the pregnant woman. It is classified as early CS - when the clinical manifestations occur in the two first years of life - or late CS - when the manifestations occur after the second year. The infection can cause severe consequences for the fetus: abortion, fetal death and motor, cognitive, neurological, visual, and auditory sequels. Vertical transmission is preventable, as long as the woman is diagnosed early and treated properly.^3^

Despite being an old, well-known disease, with established low-cost diagnosis and treatment, CS is still considered by the World Health Organization (WHO) as a public health issue. In 2017, the United Nation Children’s Fund (UNICEF) and the Pan American Health Organization (PAHO) determined a goal to reduce the incidence of CS in Latin America to 0.5 cases/1,000 live births (LB) until 2015.^4^

The most recent Brazilian data, expressed in the 2016 Syphilis Epidemiological Bulletin, showed not only the failure to reach that goal, but also the increasing incidence rates and infant mortality because of that disease. The incidence of CS in children younger than 1 year of age rose from 1.7 cases/1,000 LB in 2004 to 6.5 cases/1,000 LB in 2015. Childhood mortality caused by syphilis passed from 2.4/100 thousand LB in 2005 to 7.4/100 thousand LB in 2015. In the state of São Paulo, 24,108 cases of CS were notified from 1987 to 2015 (until June 30, 2015), and, in 2015, the incidence rate in children younger than 1 year of age was of 5.9 cases/1,000 LB.^5^

The WHO estimates an incidence of 12 million new cases of syphilis per year around the world, including 1 million pregnant women. In the United States, the prevalence of CS increased 27.5% between 2013 and 2014, reaching 11.6 cases/100,000 LB in 2014. Even in developed countries, the infection with syphilis during pregnancy is still a significant cause of stillbirths and infant morbidity.^5^ ^,^ ^6^ ^,^ ^7^ Besides, it is not rare that opportunities of preventing the infection and the sickening of children because of CS are missed. Therefore, it is important to stay alert for possible flaws in the strategies of prevention, both in basic care and in the secondary and tertiary levels.

Therefore, the objective of this study was to describe a case of CS with severe clinical manifestations and late diagnosis, and to identify the flaws in the strategies of prevention of this disease in several stages/levels of healthcare, which led to the delayed diagnosis.

## CASE DESCRIPTION

A boy aged 34 days, born in the city and Metropolitan region of the state of São Paulo, referred from the Basic Health Unit (UBS) to Hospital de Clínicas of Universidade Estadual de Campinas (HC Unicamp), for presenting with increased abdominal volume and progressive jaundice for 2 weeks, besides fecal hypocholia in the past week. The following exams had been performed: hemoglobin (Hb): 8.1 g/dL, platelets: 85,000/mm^3^, total bilirubin (TB): 13.3 mg/dL, direct bilirubin (DB): 8.0 mg/dL, aspartate aminotransferase (AST): 220 U/L, alanine aminotransferase (ALT): 119 U/L, alkaline phosphatase (ALP): 684 U/L. Abdominal ultrasound: contracted biliary vesicle, hepatosplenomegaly, small ascites and thick-walled bowel loops. The mother reported reddish lesions with vesicles, blisters and desquamation at the palms and soles since birth.

Gestational history: Third pregnancy of the mother, with history of one previous spontaneous abortion and one living healthy child. Prenatal care with six appointments, negative serology for HIV, syphilis and hepatitis in the first trimester, not repeated afterwards. The mother reported use of drugs (amphetamine, alcohol and cocaine), during the pregnancy. The children was born of natural birth, at the hospital, weighing 3,000 g, measuring 48 cm, cephalic perimeter of 35 cm, Apgar score at 1 and 5 minutes of 9 and 10, and gestational age assessed by the physical examination of 37 weeks and 2 days. He was discharged from the maternity ward with 48 hours of life. In the child’s birth card, as well as in the mother’s prenatal card, there were no data about the performance of maternal serology for syphilis at the time of birth. Afterwards, after contacting the maternity ward, the information of maternal nonreactive result for VDRL (Venereal Diseases Research Laboratory) during the hospitalization for the delivery was received. A treponemal test was not carried out at the time. Serology for syphilis was not performed in the child.

**Figure 1:**
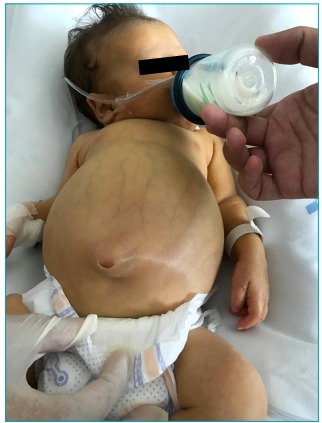
Picture of the patient at admission in Hospital de Clínicas at Universidade Estadual de Campinas.

**Figure 2:**
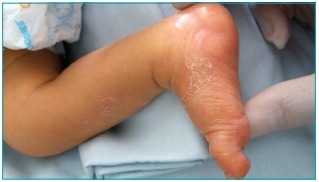
Picture of the patient at admission in Hospital de Clínicas at Universidade Estadual de Campinas.

**Figure 3:**
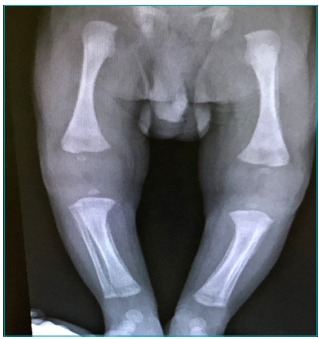
Characteristic periostitis periosteal thickening, affecting femur, and tibia, bilaterally.

At the physical examination for the hospitalization, the child was weighing 3,680 g and cephalic perimeter of 35 cm. There were erythematous desquamative lesions in the hands and feet, and macular exanthema in the inguinal region. At the physical examination, the following were identified: ascites, ++/4+ jaundice, palpable liver 5 cm below the right costal and palpable spleen 3 cm from the left costal margin (Figures 1 and 2). Laboratory exams: Hb: 7.3 g/dL, Platelets: 20,000/mm^3^, TB: 16.7 mg/dL, DB: 14.8 mg/dL, AST: 244 U/L, ALT: 105/U/L, Gamma-glutamyltransferase (GGT): 95 U/L, ALP: 539 U/L, INR: 1,24, R: 2.23. Syphilis serology: reactive CMIA (chemiluminescent microparticle immunoassay), VDLR: 1:1024, reactive TPHA (*Treponema pallidum Hemaglutination*), non-reactive HIV, toxoplasmosis, hepatites B and C and cytomegalovirus serologies. Cephalorachidian fluid was not collected due to low platelet cont. The long-bone x-ray showed symmetric and disseminated periostitis, lucent metaphyseal bands and periosteal thickening of femur, humerus, ulna and tibia (Figure 3). Transfontanelar ultrasound and fundus oculi showed no changes. Maternal serology for syphilis (collected right after the positive result of the child): reactive CMIA and TPHA, VDRL 1:256. Other maternal serologies were negative. Paternal serology for syphilis was also reactive, performed in a UBS right after the diagnosis of the child. The father did not provide the result of the staff, nor the results of other serologies. The father reported the use of psychoactive substances.

A treatment with intravenous crystalline penicillin, 50,000 UI/kg/dose of 4/4 hours per 10 days was started, as established for neurosyphilis by the Ministry of Health (MH). During the treatment, the child received a concentrate of red blood cells and supplementary offer of oxygen, since he presented with respiratory distress due to the restrictive effect caused by large ascites. He improved, both clinically and in laboratory terms: Hb: 10.9 g/dL, Platelets: 89,000/mm^3^, TB: 12.85 mg/dL, DB: 8.1 mg/dL, AST: 244 U/L, ALT: 105 U/L, GGT: 182 U/L, ALP: 634 U/L, INR: 1.66, R: 1.09. At this moment, there was an attempt to collect cerebrospinal fluid, however, without success due to technical difficulties. He was discharged on the 18th hospitalization day, for outpatient clinic follow-up in the pediatric gastroenterology and infectology medical clinics. During hospitalization, the mother began a treatment with 3 weekly doses of 2,400,000 UI of benzathine penicillin, and the father was referred to treatment and follow-up in the UBS.

## DISCUSSION

In the case reported, an opportunity to diagnose CS during pregnancy, in the hospitalization for birth and in the neonatal unit was missed, because of the following facts: there was no maternal serology performed for syphilis in the last trimester; the clinical manifestations of the child at birth were not investigated; and the treponemal test was not performed in the hospitalization for the birth. Only the nontreponemal test (VDRL) of the mother took place, and had a false-negative result, probably due to the prozone effect.^8^ In hospitalization for birth, or even for curettage after abortion, a nontreponemal test must be carried out (VDRL), associated to a treponemal test, regardless of the results of the serologies performed in the prenatal period. The fast test is used in the UBS to rapidly identify pregnant women who have had previous contact with treponema, with high risk of presenting untreated syphilis. The fast test can be used at the time of delivery, as long as accompanied by the VDRL.^9^

The importance of conducting the treponemal test with the VDRL is owed to the possibility of false-negative results in the nontreponemal test. False-negative results can occur in the initial stage of the disease, in late latent syphilis and in late syphilis, as well as a result of the prozone effect. The prozone effect takes place especially at the recent stage of syphilis or during pregnancy. It happens when there are excessive antibodies in the tested serum, which leads to the blockage of antigens and to the inhibition of the reaction of the test, leading to a false-negative result. To prevent that from happening, it is important to dilute the tested sample, until 1:4 or 1:8. In general, Brazilian laboratories perform the VDRL test to diagnose syphilis in the 1:1 dilution, which enable the occurrence of this event.^8^

It is worth to point out the importance of the placenta at the time of delivery, because placentitis caused by *Treponema pallidum* is clinically presented by a pale, crude, large placenta. In these cases, the placenta must be sent for a histopathological test, to investigate the syphilis diagnosis.^9^

Even though approximately 70% of the newborns are asymptomatic at birth, it is known that CS can lead to clinical manifestations in the first days of life, or even at birth, and can be identified in the first physical examination - which takes place still in the maternity ward.^10^ In this case, there was a flaw also in the physical examination of the newborn, once he already presented with vesicles, blisters and desquamation in the hands and feet, typical aspects of the disease in its congenital form, according to the mother’s information. After discharge, the newborn attended a child care appointment in a UBS, in which new clinical manifestations were identified, such as jaundice and increased abdominal volume. Still, the child’s diagnosis only happened after the referral to a tertiary service to investigate for the cholestatic syndrome.

From 1998 to June 2016, the Health Information System (SINAN) received 142,961 notifications of CS cases in children younger than 1 year. In 2015, 19,228 cases were notified, and, of these, 18,938 (98.1%) were diagnosed in the neonatal period, of which 96.4%, in the first week of life.^5^ Considering these levels, the conclusion is that this case was diagnosed late in comparison to most Brazilian cases, and, consequently, the treatment was administered late, which means higher morbidity and higher risks of sequels for the patient.

Lago and Garcia^11^ described, in 2000, three cases of infants with late diagnosis of syphilis, who needed hospitalization via the emergency room, aiming at warning the emergency services of the importance of suspecting this diagnosis, since it can pass unnoticed in the neonatal period. This study shows the importance of this warning even 17 years later, because despite the development of preventive programs and strategies, in face of the current prevalence and possible flaws in the prevention and early detection of the disease, this hypothesis should be included in the differential diagnosis of the daily cases in the emergency services.

It is known that the occurrence of so many cases of CS, even in the metropolitan regions - with more access, and, supposedly, better quality in women and children health care -, shows serious problems in prenatal care. These flaws can be attributed to problems in coverage and quality of the prenatal care, in the diagnosis and treatment of basic care, in the collection and treatment of sexual partners and in the follow-up of the treatment.^9^ However, it is important to highlight that there may be flaws in the early diagnosis of CS in the maternity wards or in the first child care appointments - as is this case -, therefore mentioning the importance of all newborns being discharged from the maternity ward only after the result of maternal serology - treponemal test associated to a nontreponemal test -, negative for syphilis.

In epidemiology, the term “sentinel event” is used to name events involving problems or preventable deaths, possibly associated with the poor quality of the preventive or therapeutic interventions, working as a warning to health professionals that these strategies must be improved.^12^ Flaws in prevention barriers in different levels of health care identified in this case characterizes a sentinel event. 

The preventive measures for CS considered to be efficient are established in mandatory stages according to the MH. First, all pregnant women must have access to qualified prenatal care, considered sufficient when she has at least six appointments. The Operational Plan for the Reduction of HIV and Syphilis Vertical Transmission, from 2007, established the serological screening of all pregnant women twice, in the first and in the third trimesters. In case the infection takes place during pregnancy, it can be identified in the last trimester.^10^ In the described case, it is believed that the woman contracted the infection after the first trimester; however, the lack of maternal serology in the last trimester and the false-negative result on the day of birth made the diagnosis not possible. It is worth to mention that screening during pregnancy has low cost and is easy to access, involving only a screening test, usually the nontreponemal VDRL. In cases in which VDRL is positive, more specific treponemal tests are instituted. Pregnant women who presented positive serology must be called for the immediate beginning of the treatment, as well as their partners, who must be tested with the treponemal test, or a fast test, and treated according to the current recommendations.^9^

Aiming at the early identification of pregnant women who are at risk for syphilis, and in order to ensure the early treatment during pregnancy, the MH, through Ordinance n. 1,459/GM/MS^13^, from June 24, 2011, instituted the fast test, as action of prevention and treatment of sexually transmitted diseases (STDs). This is an easy test, which does not require a laboratory infrastructure, and whose reading takes from 10 to 15 minutes. The nursing teams of the UBS were trained to execute, read and interpret the results of the fast test in pregnant women and their partners.^14^ The identification of the pregnant women with syphilis during prenatal care allows the treatment, which, when conducted properly in the first trimester, prevents the fetus infection. In order to be considered properly treated, the woman must present: treatment with adequate doses of benzathine penicillin according to the stage of the disease, documented in the prenatal card, and concluded at least 30 days before labor, besides the verification of the partner’s treatment, simultaneously. The treatment of recent syphilis in the pregnant women (primary, secondary and early latent stages) involves two series of 2,400,000 UI of penicillin G benzathine, with a 1-week interval between the doses. In the late latent and tertiary stages, or unknown, the scheme involves 3 series of 2,400,000 UI. There must also be the verification of a twofold decrease in nontreponemal titer at the time of birth, or stable titers if the initial titer was lower than or equal to 1:4.^3^ ^,^ ^9^

Among the cases of CS notified in 2015, 78.4% of the mothers had underwent prenatal care. Of these, 51.4% were diagnosed with syphilis during pregnancy, and 34.6%, at the time of delivery / curettage.^5^ These numbers, in accordance with the failed prenatal care performed in this case, reflect the low quality of care addressed to pregnant women in the country, and the belittling of the measures, proven to be efficient, in the diagnosis and treatment of syphilis during pregnancy.

Immediately after the hospitalization for delivery in the maternity ward, or even in cases of abortion, it is mandatory to perform a treponemal test and a nontreponemal test for syphilis. In cases of mothers diagnosed with syphilis in the positive treponemal test, there is a general evaluation of the child by requiring tests with cerebrospinal fluid, long-bone x-ray and hemogram. The cerebrospinal fluid collection is mandatory for the neurosyphilis research. When it is not possible to collect it due to low platelet count, for example, it is important to consider the possibility of neurosyphilis and continue with the recommended treatment with crystalline penicillin, as in this case. Besides, as soon as possible, the fluid must be collected. In that case, after the platelet count improves, there was an attempt to collect the cerebrospinal fluid, however, it was unsuccessful due to technical difficulties. Therefore, the child was discharged, with outpatient clinic schedule to undergo this examination.^10^ ^,^ ^15^

This clinical case involving a child with late diagnosis with syphilis points to the need of paying attention and fulfilling all the actions established by the MH. with respect to pregnant women’s and newborn’s care, with an ultimate goal of identifying and treating the disease as early as possible.
